# Sensory neuroprosthesis improves postural stability during Sensory Organization Test in lower-limb amputees

**DOI:** 10.1038/s41598-020-63936-2

**Published:** 2020-04-24

**Authors:** Hamid Charkhkar, Breanne P. Christie, Ronald J. Triolo

**Affiliations:** 1 Department of Biomedical Engineering, Case Western Reserve University, 10900 Euclid Avenue, Cleveland, OH 44106 USA; 2 Louis Stokes Cleveland Veterans Affairs Medical Center, 10701 East Boulevard, Cleveland, OH 44106 USA

**Keywords:** Somatic system, Sensory processing, Translational research, Biomedical engineering

## Abstract

To maintain postural stability, unilateral lower-limb amputees (LLAs) heavily rely on visual and vestibular inputs, and somatosensory cues from their intact leg to compensate for missing somatosensory information from the amputated limb. When any of these resources are compromised, LLAs exhibit poor balance control compared to able-bodied individuals. We hypothesized that restoring somatosensation related to the missing limb via direct activation of the sensory nerves in the residuum would improve the standing stability of LLAs. We developed a closed-loop sensory neuroprosthesis utilizing non-penetrating multi-contact cuff electrodes implanted around the residual nerves to elicit perceptions of the location and intensity of plantar pressures under the prosthetic feet of two transtibial amputees. Effects of the sensory neuroprosthesis on balance were quantified with the Sensory Organization Test and other posturographic measures of sway. In both participants, the sensory neuroprosthesis improved equilibrium and sway when somatosensation from the intact leg and visual inputs were perturbed simultaneously. One participant also showed improvement with the sensory neuroprosthesis whenever somatosensation in the intact leg was compromised via perturbations of the platform. These observations suggest the sensory feedback elicited by neural stimulation can significantly improve the standing stability of LLAs, particularly when other sensory inputs are depleted or otherwise compromised.

## Introduction

Individuals with lower limb amputation face challenges in maintaining their balance when navigating uneven terrains or encountering perturbations during walking^1–3^. The fear of falling and decreased balance confidence are prevalent among lower limb amputees (LLAs)^2,4^, which are important factors in their mobility and participation in social activities^4–8^. Compared to individuals without lower limb loss, LLAs have slower walking speeds, possibly because of decreased gait stability and the need for increased conscious attention while walking on uneven or changing terrains^1^. In a survey of community-dwelling LLAs, more than 50% reported that they had fallen at least once in the past year^4,9^. Amputees typically place more trust in the intact limb, which results in overuse and destructive long-term consequences, such as osteoarthritis of the intact knee and/or hip^10^. Decreased loading on the affected limb can also lead to osteopenia and subsequent osteoporosis. With the growing number of people who lose limbs due to vascular diseases or trauma, it is important to develop assistive technologies that improve standing stability in this population.

Three main sensory systems, the visual, vestibular, and somatosensory, contribute to stable posture during stance^11,12^. Theses inputs are integrated and processed in the central nervous system which generates appropriate movement strategies and motor commands to maintain postural stability^13,14^. However, when any of the sensory inputs are absent or inaccurate, the CNS adjusts the gains for each input to control the stability^13^. Such adjustments are often demonstrated in increased body sway, and if not successful can result in loss of balance and falls.

The absent sensory feedback from the missing foot in LLAs plays a crucial role in the degradation of their balance^15–17^. LLAs mainly rely on other sensory inputs, such as vision or proprioception from the intact and residual lower limbs, to compensate for compromised sensory information^3,17^. When vision is blocked, LLAs have significantly more postural sway and are less stable compared to able-bodied controls^18,19^, indicating that the lack of somatosensory feedback from the missing limb contributes to the marked differences in stability^17^. Moreover, unilateral amputees use sensory feedback from their intact ankle and foot to compensate for the somatosensory information lost with the missing limb. Studies show unilateral amputees rely more on their intact limb to make balance adjustments and reduce the risk of fall^20^. When LLAs have trouble maintaining balance with their intact leg, they are more likely to have poor functional outcomes related to personal care, household activities, and recreational activities^21^.

Electrical stimulation of the remaining nerves in the residual limb of LLAs via various neural interface technologies can elicit somatosensory percepts referred to the missing limb^22,23^. The modality and the intensity of the reported sensations can be modulated by tuning the stimulation parameters^23^. The sensations evoked by non-penetrating multi-contact cuff electrodes implanted on the peripheral nerves above the knee in the residual limbs of LLAs have been robust and consistent for more than two years. Furthermore, the perceived sensations generated by neural stimulation have central processing times and temporal sensitivities similar to natural tactile sensation^24^. Although LLAs report improvements in self-reported confidence with the sensory feedback elicited by neural stimulation^22,25^, the effects of such feedback on objective measures of balance has not previously been determined.

**Figure 1:**
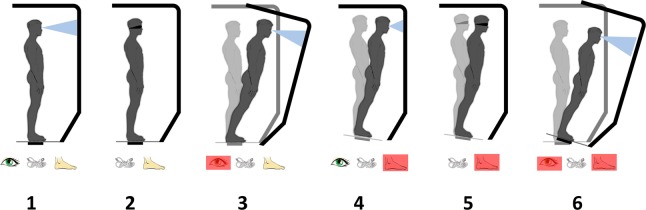
Conditions of SOT in which controlled perturbation to visual and somatosensory inputs could be applied. Red boxes denote perturbation of the corresponding sensory input. Participant’s eyes were closed in conditions 2 and 5.

The Sensory Organization Test (SOT) has been utilized as a clinical and research tool to objectively and quantitatively examine the contribution of different sensory systems to standing balance^12^. In the SOT, visual and somatosensory inputs are selectively perturbed or missing (Fig. 1) and the results on postural control are examined individually and in combination^8^. Outcomes of the test are correlated to overall balance performance during ambulation and activities of daily living^2,26^. The SOT has been administered on different patient populations with standing stability deficits, including stroke survivors^27^, individuals with Parkinson’s Disease^28,29^, LLAs^2,3^, and elderly people^14,26^.

Standing balance is one of the most basic tasks in amputee rehabilitation and plays an essential role in most functional activities^16,30^. In this study, we examined whether the sensory feedback provided by chronically implanted non-penetrating, epineural nerve cuff electrodes could improve balance stability in transtibial amputees. Our hypotheses were: (1) somatosensory feedback elicited by direct neural stimulation will reduce the sway exhibited by LLAs when other sensory inputs are perturbed, and (2) electrically elicited sensations related to the missing foot will improve weight distribution symmetry between the intact and prosthetic limbs. The results of this study may have implications to the development of new prosthetic technologies intended to reduce the risk and fear of falls, improve standing balance and balance confidence, encourage engagement in unstructured community environments, or accelerate the rehabilitation process following lower limb amputation.

## Methods

### Research participants

Two individuals with unilateral transtibial amputations (LL01 & LL02) volunteered and enrolled in this study. A summary of their characteristics at the time of enrollment is presented in Table 1. Both participants were regular prosthesis users with no medical history of peripheral neuropathy, dysvascular disease, phantom pain, or uncontrolled diabetes. Participants had no fall history for at least nine months prior to the beginning of the study, and were therefore both classified as non-fallers^2^. The experiments described in this work were conducted at least a year after their enrollment. However, during the first year post nerve cuff implantation, both participants regularly visited the laboratory where they received neural stimulation and performed other tests including impedance measurements, sensory threshold determination, sensory mapping, and psychometric experiments described elsewhere^23,31,32^. The Louis Stokes Cleveland Veterans Affairs Medical Center Institutional Review Board and Department of the Navy Human Research Protection Program approved all study procedures, which were conducted under an Investigational Device Exemption obtained from the United States Food and Drug Administration. The study was designed in accordance with relevant guidelines and regulations, and both individuals gave their written informed consent to participate.

**Table 1:** Summary of participant characteristics enrolled in the study.

Participant	Sex	Age (year)	Height (cm)	Weight(kg)	Amputated side	Etiology	Time since amputation
LL01	M	67	173	106	Left	Traumatic	48
LL02	M	54	168	67	Right	Traumatic	11

### Neural interface technology

**Figure 2:**
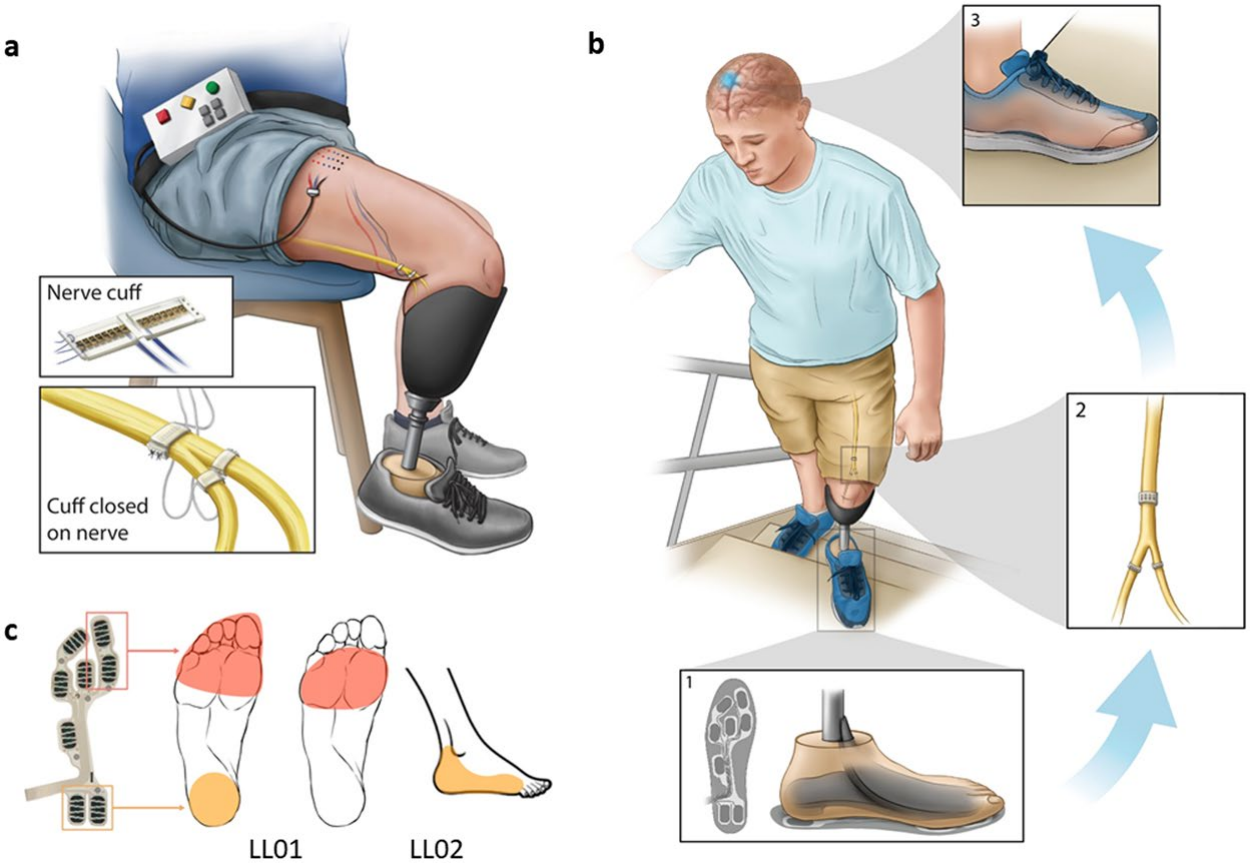
Neural interface technology and the sensory neuroprosthesis. (**a**) The cuff electrodes were implanted on sciatic and/or tibial and peroneal nerves. The access to individual contacts within each cuff electrode was through percutaneous leads, which connected to an external stimulator. (**b**) Pressure distribution under the prosthetic foot is sensed via an array of FSRs integrated into an in-shoe insole, electrical stimulation to specific cuff contacts is determined based on the FSR readings, and sensations that match in location and perceived intensity of the pressure profile under prosthetic foot are elicited. (**c**) Reported percept locations from LL01 and LL02. Stimulation delivered selectively to contacts generating perceived sensations referred to the missing toes and heels in response to pressures applied to the insole FSR array.

The details of neural interface technology and implantation technique have been described previously^23^. Both participants had 16-contact Composite Flat Interface Nerve Electrodes (C-FINEs) installed around their sciatic, tibial and/or common peroneal nerves during an outpatient surgical procedure. All C-FINE contacts were connected to percutaneous leads via industry-standard 8-contact in-line connectors (Medtronic Inc.). The percutaneous leads exited the skin on the upper anterior thigh. To deliver stimulating currents during laboratory visits, the percutaneous leads from C-FINEs were connected to a custom-designed external stimulator^23,32^. Figure 2a depicts schematically the implanted and external components of the system.

### Electrical stimulation

The pulse amplitude range for the external stimulator was 0–5.6 mA with the resolution of 0.1 and 0.2 mA for values below and above 2 mA, respectively. The pulse width (PW) could be modulated between 0–255 μs with a resolution of 1 μs^23,33^. Stimulating currents were delivered to the nerves in a series of asymmetric, charge-balanced, cathodic-first pulses with return to a common anode placed on the skin above the iliac crest. Stimulation parameters were set through a custom-made routine in Simulink (MathWorks Inc.) and then compiled and downloaded into a dedicated computer running xPC Target real-time kernel (MathWorks Inc.) for real-time operation during standing experiments. An optical isolator between the xPC target computer and the stimulator ensured electrical isolation between the participant and other AC-powered electrical equipment. Stimulation charge density was kept below 60 uC/cm^2^ to avoid any potential of damage to the neural tissue and/or platinum contacts^23,31^.

### Sensory neuroprosthesis

Able-bodied individuals sense their center of pressure partly through cutaneous sensation from the plantar surface of the foot. The pressure distribution under the feet changes as they sway in an anterior-posterior or medial-lateral direction, which provides feedback utilized by the central nervous system to maintain balance. Similarly, we implemented a mechanism of sensory feedback in which the perceived intensity of elicited sensations was proportional to pressure underneath the prosthetic foot (Fig. 2b). For each participant, stimulating currents were delivered through a subgroup of C-FINE contacts to elicit sensations corresponding to pressures applied to either the heel or forefoot (Fig. 2c). This selection was based on prior mapping experiments^23^.

The pressure distribution underneath the prosthetic foot was measured using dynamic force-sensing resistors (FSRs) incorporated into a shoe insole (IEE S.A.). The resistance for the FSRs was more than 1 MΩ when the insole was unloaded and decreased to 2 KΩ with increasing loads up to 70 N/cm^2^ pressure. Each insole contained eight individual FSR cells, and readings from cells were collected using a data acquisition board (NI PCI-6071E, National Instruments) with a sampling rate of 1000 Hz. The readings from two FSR cells at the heel were averaged together to estimate an overall value for rearfoot load. Similarly, readings from the first metatarsal and the big toe FSR cells were averaged together to provide an estimate of the overall load on the forefoot.

To modulate the perceived intensity of the elicited sensation, stimulation PW varied proportionally in response to pressure readings from the FSR insoles. A calibration process was performed to determine the minimum, reference, and maximum FSR values and their corresponding PWs. Minimum values were recorded when no load was applied to the insole by the prosthetic foot (i.e., either while subjects sat or stood with their prosthetic foot off the ground), which were associated with sub-sensory threshold PWs. Reference values were obtained by having participants stand with equal weight on both legs, and the corresponding PWs were set such that the perceived intensities matched the pressures reported for the intact foot. Isolated maximal pressures were then applied to the forefoot and rearfoot by shifting body weight to the prosthetic toe and heel, respectively, and recording the maximum signal values from the corresponding regions of the FSR insole. In these postures, PW values were set at levels that participants verbally confirmed were higher in perceived intensity than the sensations elicited with the prosthetic foot flat on the ground in the reference position. Having established the minimum, reference, and maximum values for the FSR and PW values, the input-output relationship between pressure and PW was defined by a piecewise linear function (Eq. 1):

1 $$pw(v)=\{\begin{array}{c}\,0,\,v < {V}_{min}\\ \left(\frac{P{W}_{ref}-P{W}_{min}}{{V}_{ref}-{V}_{min}}\right)v,\,{V}_{min}\le v\le {V}_{ref}\\ \left(\frac{P{W}_{max}-P{W}_{ref}}{{V}_{max}-{V}_{ref}}\right)v,\,{V}_{ref}\le v\le {V}_{max}\\ P{W}_{max},\,{V}_{max}\le v\end{array}$$

where the *v* is the voltage readings from FSR.

### Experimental design

The SOT was administered using a SMART Balance Master (Natus Medical Inc.). The device was equipped with a controllable platform with two embedded dynamic force plates capable of anterior-posterior translation or rotating about the ankle, and a visual surround capable of rotating about the subject. Movements of the platform and visual surroundings were controlled by the NeuroCom Balance Manager Software Suite (Natus Medical Inc.). Participants were tested under six sensory conditions while they were secured in a loosely fitting safety harness attached to an overhead bar. The conditions for the SOT, as listed in Table 2 and illustrated in Fig. 1, involve visual and/or somatosensory perturbations. Rotations of the platform and/or the visual surroundings in the fore-aft direction was proportionally matched with a gain to the sway of each participant during the test, such that higher postural sway resulted in greater perturbations in the platform or visual surroundings. The gain was selected after test trials in which participants found it difficult to maintain their balance during the most challenging condition (#6 – inaccurate visual and compromised somatosensory inputs), yet not to a degree that it would result in a fall. Prior work with lower-limb amputees performing the SOT either excluded trials with falls in data analysis^34^ or allowed participants to repeat the trial^8^, which could skew the sway-related outcomes. Therefore, we decided to set the gain such that the test would be maximally challenging without compromising the validity of the analysis. For LL01 and LL02 the gains were set to 1 and 2, respectively.

**Table 2:** Summary of conditions in SOT.

Condition	Platform	Eyes	Surrounding
1	Stationary	Open	Stationary
2	Stationary	Closed	Stationary
3	Stationary	Open	Moving
4	Moving	Open	Stationary
5	Moving	Closed	Stationary
6	Moving	Open	Moving

Each SOT condition lasted for 20 s and participants were instructed to maintain as little postural sway as possible, to keep their feet in the same position throughout the test, and to keep their arms at their sides. One test block consisted of all six SOT conditions, with each condition tested two times. Each block was performed under one sensory stimulation mode: closed-loop sensory neuroprosthesis active (stimulation “on”) or inactive (stimulation “off”). The order of conditions was randomized within each block, and the order of stimulation modes was randomized between blocks. Six blocks were collected for each sensory stimulation mode in total, i.e. 12 trials for each SOT condition and sensory stimulation mode.

For every trial, the time series of ground reaction forces, Center of Pressure (COP), and estimates of Center of Gravity (COG) were extracted using the clinical module in the NeuroCom Balance Manager Software Suite. The raw force plate data were sampled at 100 Hz and saved on a local hard drive for offline processing.

### Data analysis and outcome measures

Equilibrium Score (ES), a clinically known measure to quantify sway amplitude during SOT conditions^3^, was calculated for every trial based on Eqs. 2 and 3, consistent with the built-in equations used in NeuroCom Software Suite clinical module^35,36^.

2 $$ES=100\times \frac{{12.5}^{o}-(Max({\theta }_{A})-Max({\theta }_{P}))}{{12.5}^{o}}$$

In Eq. 2, *Max*(*θ*_*A*_) and *Max*(*θ*_*P*_) are the maximum COG angular sways in the anterior and posterior directions, respectively. 12.5^o^ is an accepted range of anterior to posterior sway before an able-bodied individual loses balance during stance^37,38^. An ES approaching 100 denotes minimal sway, whereas scores around zero indicate that balance is approaching the limits of stability. The *Max*(*θ*_*A or P*_) was calculated using Eq. 3, in which *h* is the participant’s height^36^:

3 $$Max({\theta }_{AorP})(deg)={\tan }^{-1}\left(\frac{Max(CO{G}_{AorP})(cm)}{0.55\times h(cm)}\right)\times \frac{{180}^{o}}{\pi }$$

Because ES only considers extreme limits of sway angle, it cannot capture the complete sway history during a trial. Therefore, we calculated two additional sway-related outcomes, Root Mean Square (RMS) distance of the COP, and elliptic area approximation of COP. In summary, the higher ES indicates better balance. Conversely, higher values for COP-related measures indicate less stability.

The RMS distance of the COP (*DIST*_*RMS*_) is an indicator of variability in COP movement. It has been shown to be a reliable measure of postural equilibrium^39–41^ and is sensitive to altered sensory inputs^42,43^. *DIST*_*RMS*_ was calculated using Eq. 4:

4 $$DIS{T}_{RMS}=\sqrt{\frac{1}{N}\mathop{\sum }\limits_{1}^{N}RD{[n]}^{2}}$$

where N is the total number of samples during a trial, N = 2000 and *RD*[*n*] is the resultant distance (RD) vector of the COP as given below (Eq. 5):

5 $$RD[n]=\sqrt{CO{P}_{AP}{[n]}^{2}+CO{P}_{ML}{[n]}^{2}}$$

In Eq. 5, COP_AP_ and COP_ML_ are the COP components in the Anterior-Posterior (AP) and Medial-Lateral (ML) directions, respectively. The lower-case ‘n’ in Eqs. 4 and 5 indicates a discrete-time sample. The mean values of the AP and ML components were subtracted from the COP vectors in every trial to eliminate any inconsistency due to foot placement across trials. The mean was calculated over the 20 s, the period of the trial.

Additionally, an elliptic area approximation of the COP path was computed for each trial. This measure captures the changes in COP path during standing and has been utilized as an indicator of overall postural performance^44^. Following the method described in Schubert *et al*.^45^, we calculated a 95% prediction ellipse based on the assumption that points in the COP scatter follow a Chi-square distribution^45^. The area of the ellipse was calculated using Eq. 6:

6 $$Are{a}_{PE}=\pi ab$$

where a and b were semimajor and semiminor axes of the confidence ellipse and they were estimated according to Equation 7:

7 $$a=\sqrt{{\chi }_{2}^{2}.{\lambda }_{1}},b=\sqrt{{\chi }_{2}^{2}.{\lambda }_{2}}$$

In Eq. 7, λ_1_ and λ_2_ are eigen values of the COP covariance matrix. ${\chi }_{2}^{2}$ is the value of the Chi-square cumulative distribution with two degrees of freedom at probability level = 0.95.

Lastly, any changes in weight symmetry were ascertained by calculating the percentage of the body weight placed on the prosthesis. The ground reaction forces from the force plate underneath the prosthetic foot were normalized to the sum of ground reaction forces from both feet, i.e., body weight.

### Statistical analyses

A two-way ANOVA was conducted to examine the effects of stimulation mode (i.e., sensory neuroprosthesis active or inactive) and SOT condition on the means ± standard deviations of the outcome measures. Extreme outliers, defined as data points more than three interquartile ranges away from either the lower quartile or upper quartile, were removed from the analysis. Normality was assessed using Kolmogrov-Smirnov normality test for each cell of the design. Any statistically significant interactions between stimulation mode and SOT conditions were followed up by analysis of simple main effects to determine the impact of stimulation under specific SOT conditions. For the analysis of simple main effects, the statistical significance received a Bonferroni adjustment for the two stimulation modes and was accepted at the p < 0.025 level. If no interaction effects were found, we tested for the main effect of stimulation on the measured outcome. Because there were only two stimulation modes, no post hoc analyses were deemed necessary. For all other comparisons, we used two-tailed t-tests followed by Bonferroni adjustments if multiple paired comparisons took place. All the statistical analysis were performed using IBM SPSS Statistics Ver. 22 (IBM Corp.).

## Results

### Sensations elicited by the sensory neuroprosthesis

For both participants, stimulation was delivered via different contacts in the cuff electrodes implanted on the sciatic nerve. For LL01, when pressure was applied to the FSRs at a location corresponding to the first metatarsal and big toe of the prosthetic foot, electrical stimulation was delivered to evoke sensations perceived as arising from the missing forefoot (Fig. 2c). The readings from the first metatarsal and the big toe FSR cells were averaged together to provide an estimate of the overall load on the forefoot. In response to pressure on the FSRs underneath the prosthetic heel, the neuroprosthesis elicited sensation perceived as originating in the missing heel. The readings from two FSR cells at the heel were averaged together to estimate an overall value for the rearfoot load.

Similarly, for LL02, pressure to the first metatarsal and toe FSRs triggered electrical stimulation, which elicited sensation related to the missing first to fifth metatarsal areas. In response to pressure on the heel FSRs, the neuroprosthesis elicited sensation perceived as arising from the missing heel and lateral ankle.

### Effects of sensory stimulation on ES

**Figure 3:**
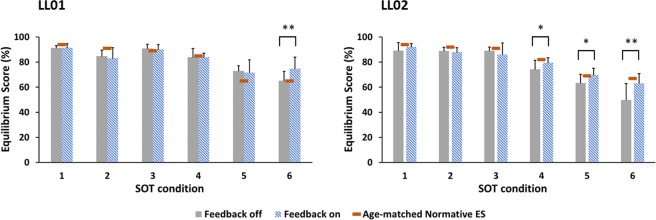
Effects of sensory stimulation on Equilibrium Score for LL01 (left) and LL02 (right). Age-matched normative means are shown in red. There was a significant interaction between stimulation mode and SOT condition. For LL02, the ES was improved with sensory feedback in conditions four, five, and six. For LL01, this improvement was observed in condition six. * and ** denote p < 0.05 and p < 0.001, respectively.

A significant interaction between stimulation mode and SOT condition for ES was found in both participants (LL01: p = 0.029, LL02: p < 0.001). This suggests that the effect of stimulation on ES depended on SOT condition (Fig. 3). For both participants, ES was significantly lower in condition six (visual and somatosensory inputs compromised) during trials with the sensory neuroprosthesis active. For participant LL02, sensory feedback also led to an improvement in ES for conditions four (somatosensation compromised) and five (vision and somatosensation compromised).

In SOT condition six, the ES values were 74.8 ± 9.6 and 65.2 ± 7.9 for the sensory neuroprosthesis active and inactive, respectively, for LL01. This represents a statistically significant mean improvement of 9.2 with 95% Confidence Interval (CI) between 5.4 to 12.9 (p < 0.001). For LL02, the ES significantly improved in conditions four, five, and six. Additionally, for LL02, the effect of electrically elicited sensory feedback on ES grew bigger from condition four to six, and approached able-bodied norms. In condition four, the ES values for LL02 were 79.6 ± 4.0 and 74.3 ± 7.5 for sensory stimulation on and off, respectively, a statistically significant mean improvement of 5.2 (95% CI 0.2 to 10.3, p = 0.042). In condition five, the ES values for LL02 were 96.7 ± 5.7 and 63.4 ± 7.1 for the sensory neuroprosthesis active and inactive, respectively, a statistically significant mean improvement of 6.3 (95% CI 1.3 to 11.3, p = 0.015). In condition six, the ES values for LL02 were 65.3 ± 4.0 and 49.7 ± 13.7 for sensory stimulation on and off, respectively, a statistically significant mean improvement of 15.6 (95% CI 10.6 to 20.7, p < 0.001).

There was a statistical difference in baseline ES values without electrically elicited sensory feedback between LL01 and LL02 in conditions four, five, and six. In condition four, without sensory stimulation, the ES values were 84.0 ± 6.8 and 74.4 ± 7.1 for LL01 and LL02, respectively (p = 0.006). In condition five, they were 72.9 ± 4.1 and 63.4 ± 6.8 for LL01 and LL02, respectively (p = 0.001). In condition six, they were 65.1 ± 7.5 and 49.7 ± 13.1 for LL01 and LL02, respectively (p = 0.004). Such differences between the two participants suggest that without the sensory neuroprosthesis active, LL01 had higher postural stability than LL02 in the last three conditions of the SOT. Lastly, the ES from these two participants were compared to previously reported aged-matched normative ES values^46^. For conditions four, five, and six, LL02 had significantly lower ES without sensory stimulation (p < 0.05) whereas LL01 either scored equal or higher than normative values (Fig. 3).

### Effects of sensory stimulation on RMS distance of COP

**Figure 4:**
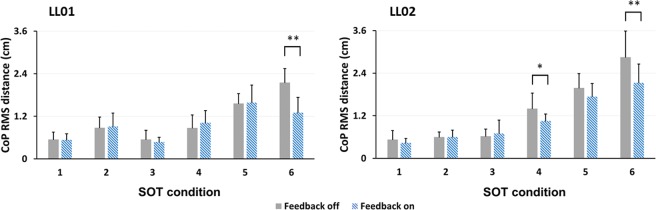
Effects of sensory stimulation on RMS distance of COP for LL01 (left) and LL02 (right). There was a significant interaction between stimulation mode and SOT condition. For LL01, the RMS distance of the COP was reduced with sensory feedback in condition six, indicating improved balance. For LL02, the reduction in RMS distance of COP was observed in conditions four and six. * and ** denote p < 0.05 and p < 0.001, respectively.

Sensory stimulation affected the *DIST*_*RMS *_in both participants, as shown in Fig. 4. For both LL01 and LL02, there was a statistically significant interaction between stimulation mode and SOT condition on *DIST*_*RMS*_ (LL01: p < 0.001, LL02: p = 0.001). For both participants, *DIST*_*RMS *_was significantly lower in condition six during trials with electrically elicited sensory feedback. For LL02 only, *DIST*_*RMS *_also improved with sensory stimulation in condition four.

For LL01 in SOT condition six, the *DIST*_*RMS *_were 1.3 ± 0.4 cm and 2.1 ± 0.4 cm for the sensory neuroprosthesis active and inactive, respectively, a statistically significant mean difference of 0.8 cm (95% CI 0.6 cm to 1.1 cm, p < 0.001). Similarly, for LL02, the *DIST*_*RMS *_during condition six were 2.0 ± 0.4 cm and 2.9 ± 0.7 cm for sensory stimulation on and off, respectively, a statistically significant mean difference of 0.8 cm (95% CI 0.5 cm to 1.1 cm, p < 0.001). Additionally, for LL02, the *DIST*_*RMS *_in condition four was 1.1 ± 0.2 cm and 1.4 ± 0.4 cm for electrically elicited sensory feedback on and off, respectively, a statistically significant mean difference of 0.3 cm (95% CI 0.1 cm to 0.6 cm, p = 0.018). In condition four, the RMS distances without sensory feedback were 0.9 ± 0.4 cm and 1.4 ± 0.4 cm for LL01 and LL02, respectively. This suggests that without sensory stimulation, LL02 had higher sway compared to LL01 (p=0.008) which might have contributed to the sensory stimulation effect seen for LL02 in condition four.

### Effects of sensory stimulation on area of prediction ellipse

**Figure 5:**
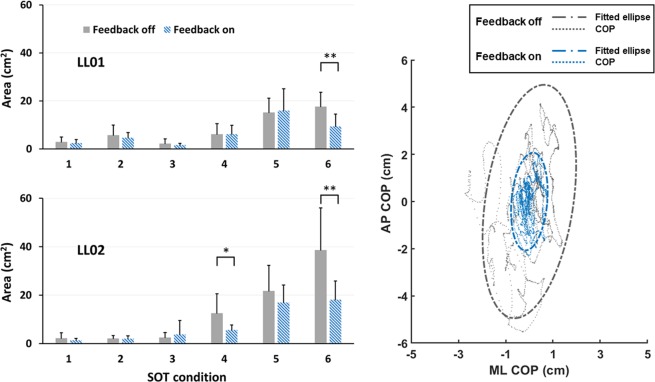
Effects of sensory stimulation on area of prediction ellipse for LL01 (top left) and LL02 (bottom left). Representative COPs and corresponding prediction ellipses from SOT condition four are shown on the right (data from LL02). There was a significant interaction between stimulation mode and SOT condition. For LL01, the area of prediction ellipse was reduced with sensory feedback in condition six, suggesting an improvement in balance. For LL02, the reduction in the area of prediction ellipse was observed in conditions four and six. * and ** denote p < 0.05 and p < 0.001, respectively.

We also found statistically significant interactions between stimulation mode and SOT condition for the area of prediction ellipse in both participants (LL01: p = 0.003; LL02: p < 0.001) that paralleled those for *DIST*_*RMS*_ (Fig. 5). For both participants, the area was significantly lower in condition six during trials with the sensory neuroprosthesis active. For participant LL02, electrically elicited sensory feedback also led to an improvement in condition four. Representative COPs and corresponding prediction ellipses are shown in Fig. 5.

In SOT condition six for LL01, the areas of the prediction ellipses were 9.3 ± 5.4 cm^2^ and 18.5 ± 2.0 cm^2^ for the sensory neuroprosthesis active and inactive, respectively. This represents a statistically significant mean difference of 9.2 cm^2^ (95% CI 5.4 cm^2^ to 12.9 cm^2^, p < 0.001). Similarly, for LL02 in condition six, the mean areas of the prediction ellipses were 18.1 ± 6.4 cm^2^ and 38.6 ± 18.2 cm^2^ for electrically elicited sensory feedback on and off, respectively. This represents a statistically significant mean difference of 20.5cm^2^ (95% CI 14.6 cm^2^ to 26.4 cm^2^, p < 0.001). In addition to condition six, LL02 exhibited a significant difference in prediction ellipse area for condition four. In this condition the mean areas of the prediction ellipses were 5.6 ± 2.2 cm^2^ and 12.5 ± 8.4 cm^2^ for sensory stimulation on and off modes, respectively, with a statistically significant mean difference of 6.93 cm^2^ (95% CI 1.03 cm^2^ to 12.84 cm^2^, p = 0.027). Without the sensory neuroprosthesis active, the area of prediction ellipse under SOT condition four was 6.1 ± 4.3 cm^2^ and 1.4 ± 0.4 cm^2^ for LL01 and LL02, respectively. This suggests LL02 had much higher fluctuations in his sway compared to LL01 (p = 0.01) without sensory stimulation.

### Effects of sensory stimulation on weight symmetry

**Figure 6:**
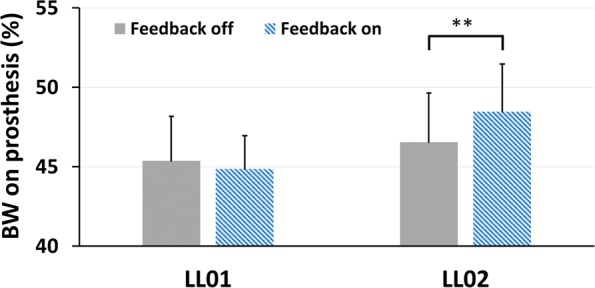
Overall effects of sensory stimulation on weight symmetry across all SOT conditions. No significant interaction between stimulation mode and SOT condition were found on weight symmetry. However, there was a statistically significant effect of stimulation on weight symmetry regardless of SOT condition for LL02. ** denote p < 0.001. In a study with 22 unilateral transtibial amputees aged 50 years or older, weight distribution during quiet stance was reported to be 44.4 ± 7.7% on the prosthetic limb^19^.

There was no statistically significant interaction between stimulation mode and SOT condition on body weight percentage on the prosthesis (LL01: p = 0.809; LL02: p = 0.571). However, the follow up analysis of the main effect for stimulation revealed a statistically significant effect of stimulation across all conditions. During trials with the sensory neuroprosthesis active, participant LL02 increased the percentage of his body weight on the prosthesis by 2% (95% CI 1.1% to 2.8%, p < 0.001) (Fig. 6). These results suggest that LL02 shifted more weight onto his prosthesis when he received sensory stimulation regardless of SOT condition. The follow up analysis of the main effects did not show any changes in body weight distribution between sensory stimulation modes for LL01 (p = 0.22). Compared to previously reported results with transtibial amputees aged 50 and older, our participants had a similar weight distribution on their prosthetic limbs^19^.

## Discussion

In this study, we demonstrated that sensations elicited in the missing foot of two transtibial amputees could decrease sway and improve balance when visual and vestibular inputs were incongruent and somatosensation in the intact foot was compromised. The sensations in the missing foot were elicited using a sensory neuroprosthesis that electrically activated nerves in the residual limb via implanted non-penetrating nerve cuff electrodes. The location and intensity of perceived sensations were determined and modulated according to prosthetic foot-floor contact pressure. Using this approach, we were able to examine the role of plantar somatosensory feedback from the missing foot during standing balance under challenging, dynamic conditions.

In both participants, we observed that the information from the sensory neuroprosthesis was most useful during condition six of the SOT, during which vestibular and visual inputs were incongruent and somatosensation in the intact leg was simultaneously perturbed. This improvement was seen in all three balance measures (ES, *DIST*_*RMS*_, and area of predicted ellipse), which demonstrates that not only were the maximum boundaries of sway reduced, but participants also remained steadier throughout the entire trial period with the neuroprosthesis active. Consistent with previous reports of naturally occurring sensory inputs, our findings show that participants utilized the most reliable sources of sensory information when others were compromised^47^, including the perceptions of plantar sensation elicited by neural stimulation.

Our results confirm that LLAs adapt to lack of sensory input from their missing limb in part by relying on sensation from the intact leg. For LL02, we found the ES decreased during all three conditions (#4–6) that perturbed somatosensation in the intact foot. A prior study showed that poor perception of vibration and pressure in the intact foot and ankle was associated with poor static and dynamic balance in dysvascular transtibial amputees^16^. Moreover, it has been reported that LLAs use their intact limb to obtain sufficient sensory information for function^16,20^. In a study by Miller *et al*., the number of reported falls per year for bilateral amputees was more than double that of unilateral amputees, suggesting that the loss of sensory input from both legs drastically increases fall risk^4^. These observations suggest that sensory neuroprostheses may be the most beneficial for LLAs with poor intact limb sensation.

The differences in the effect of the sensory neuroprosthesis on outcome measures between the two participants can be explained mainly by how they prioritized other sensory inputs. LL01 also had equal or better ES compared to age-matched able-bodied controls, an indicator of good balance stability among traumatic transtibial amputees^2,48^. Furthermore, LL01 was more stable without sensory stimulation in conditions four and five compared to LL02, which signifies that he may not have needed the additional sensory feedback as much and therefore did not utilize it in those conditions. However, LL02 found himself in a less stable situation; therefore, the electrically elicited sensory feedback resulted in an improvement in balance during the same conditions. Other factors such as residuum length^49^ and choice of prosthetic foot^6^ could have contributed to differences seen between two participants. Additionally, the amplitude for the surround and the platform movements during the SOT was chosen based on the confidence of each individual in controlling their balance. The difference in balance confidence between participants may also explain better sway measures for LL01 without the sensory neuroprosthesis active.

Maintaining balance is a complex sensorimotor function, which requires central processing of multiple sensory inputs at the vestibular nuclei^50^. The CNS compares the sensory inputs against an internal model and attributes relative weights to them to generate appropriate motor responses^51^. With reduced or conflicting sensory information, the motor performance is directly affected, and balance stability may subsequently become compromised^52^. In LLAs, not only is the sensory information from the missing foot absent, but also the internal body model has changed as a result of the altered neuromuscular and sensorimotor systems following amputation^53^. For example, it has been shown that plantar pressure sensations are used to update internal estimates of center of mass location, which is a key factor in balance stability^54,55^. It is possible that the internal models of the participants in this study were updated after the first use of the sensory neuroprosthesis. Future studies should consider baseline measurements of balance prior to providing any electrically evoked somatosensations to LLAs to investigate if updates to internal model contribute to observed improvements in balance. Alterations in the internal model by prior exposure to sensory stimulation would further support the implications that the intact neuromuscular balance control apparatus interprets the electrically elicited sensations in a similar manner to naturally occurring sensory inputs, and utilized them effectively to help maintain standing balance and stability.

The sensory neuroprosthesis appeared to improve body weight symmetry in LL02 but it did not have any significant effects on weight distribution in LL01. This finding confirms that weight symmetry in LLAs could be affected by loss of sensation, however other variables such as prosthetic alignment, prosthetic foot design, socket fit, and even poor hip abductor muscle strength could play a role in this outcome measure^19,56,57^. Moreover, several studies have shown that weight symmetry in LLAs is regained within eight weeks after first prosthesis use, and in many cases there is not much improvement beyond this period^6,58^. Since the participants were long-term prosthesis users, their no-stimulation baseline symmetry values should have stabilized. Similarly, they were both exposed to sensory stimulation in the laboratory for a year prior to these experiments, thus the symmetries exhibited with the neuroprosthesis should have also plateaued. The time course of changes in symmetry due to the sensory neuroprosthesis can be the topic of future exploration. Lastly, sensory feedback affected sway measures differently than weight symmetry, suggesting that improvements in balance are not always correlated with a more symmetrical weight distribution^6^.

It is likely that participants used the pressure exerted by the prosthetic socket on the residual limb to obtain information regarding movements of the support platform, and their own sway behaviors. However, the feedback through the socket and residuum is often not refined enough to compensate for the missing plantar sensation^16^. Additionally, sensory feedback through the socket can vary based on changes in skin sensitivity^59^, residual limb volume^60^, liner material^61^, and alignment^62^. Furthermore, in dysvascular amputees, sensation through the socket could be limited due to diminished sensation in the residual limb due to the primary disease process^48^.

In contrast to our approach that interfaces with remaining nerves in the residual limb to generate somatosensations directly referred to the missing foot, methods that utilize electro- or vibro-cutaneous input have attempted to provide indirect feedback regarding the status of the missing lower limbs^34,63–70^. However, only a few studies have examined the functional outcomes of such sensory substitution techniques with LLAs^34,67,71^. Rusaw *et al*.^34^ investigated the effects of vibratory feedback on static and dynamic balance in transtibial amputees by performing four out of the six conditions of SOT (Conditions 1–2 & 4–5). Four pressure sensors under the prosthetic foot were linked to four tactors located around the circumference of the thigh on the affected side. No improvements in any measures of sway were reported, suggesting that amputees were not able to effectively utilize the feedback functionally or integrate it into their balance control^34^. Sabolich *et al*.^66^ applied electrical stimulation to the skin of the residual limb based on the anterior and posterior loading conditions on the prosthetic foot. They reported improvement in weight distribution of transtibial amputees during static stance. However, the feedback did not result in any significant improvements in single-leg standing time, body weight symmetry, or step length symmetry during walking.

Although non-invasive approaches could be considered as preliminary tools to examine benefits of sensory feedback after limb loss, they impose limitations such as slow response time, inconsistencies based on changes in the skin-prosthesis interface, cumbersome donning and doffing, extended training times, and poor psychological acceptability and embodiment^69,71–73^. In addition to the apparent mismatch between the original and substituted sensory modality, a major limitation with sensory substitution is an abnormally long temporal delay between stimulus onset and conscious perception^73^. The response times for vibrotactile devices mounted on residual limbs of lower-limb amputees are as long as a typical gait cycle^73^, which makes this mechanism of sensory feedback impractical for balance and gait tasks that require rapid adjustments (i.e. responding to external perturbations). In addition, the detection threshold for vibrotactile or electrotactile stimuli could be greatly affected by factors such as the material of the prosthesis liner, mechanical properties of prosthetic components, skin condition, or movement of an electrode or actuator inside the socket^69,72,73^. Furthermore, because sensory substitutive approaches do not result in sensations perceived as originating in the lost limb, users must be trained to associate the external stimulus with the applied load^71^. Finally, the long-term functionality and acceptability of such systems have yet to be determined.

Our participants reported proprioception around the ankle during threshold and mapping experiments with our sensory neuroprostheses^23^, however, they do not report proprioception when postural expectations are incongruent, i.e. when standing upright with a fixed prosthetic ankle. In this scenario, the participants are consciously aware that the ankle is locked; therefore, the elicited sensations are reported as muscle tightening around the ankle or perceived as contractions of the calf muscles. Future effort will focus on integrating the sensory neuroprosthesis with volitionally controlled prosthetic ankles, so that the ankle joint is a part of the sensory neuroprosthesis and participants can benefit from elicited proprioception in addition to plantar pressure sensation.

In able-bodied individuals, three main motor strategies are utilized to maintain balance during static and dynamic conditions^3^. Movements at the ankle (i.e., the ankle strategy) are in response to small perturbations. Movements at the hip (i.e., the hip strategy) are often used to compensate for large perturbations. If there is a sudden change in the base of support in relation to the COG, then a stepping strategy is utilized to maintain balance^74^. Because transtibial amputees are missing an ankle joint, they often use the hip joint to stabilize their COG in response to small perturbations of balance^75,76^. In this case, accurate sensory feedback is still required to activate proper trunk rotation around the hip joint to maintain stability. However, if LLAs could control their prosthetic ankle joint to generate sufficient moment in response to sensory input, even greater improvements in balance could be expected from integrating a sensory neuroprosthesis with an active ankle.

Unilateral LLAs depend on visual feedback, the intact leg, and/or their upper bodies to control their posture during the early stages of rehabilitation post-amputation^53,77^. Such dependency reduces over time as they learn to capitalize on remaining sensory inputs, but amputees still primarily depend on their intact limb as well as their vision to maintain balance control during static and dynamic tasks^2,3,78–80^. Sensory neuroprostheses may have the potential to reduce dependency on these resources and accelerate progress through post-amputation rehabilitation. The results of the experiments described here were based on limited use of a sensory neuroprosthesis in the laboratory. It is possible that with continuous use of the system at home and in the community, amputees could learn to rely on the new somatosensory input and use it even more effectively in controlling balance.

Although the time since amputation for the second participant was more recent (11 years for LL02 compared to 48 years in LL01), it was unlikely that differences in SOT outcomes between participants were due to the time of amputation. A prior study conducted with 15 unilateral transtibial amputees between 2–44 years post-amputation reported no effect of the time post-amputation on SOT outcomes^8^. Both participants also received equivalent exposure to the sensory neuroprosthesis and had similar amounts and types of experiences with the system in the laboratory on a weekly basis for approximately 1.5 years prior to testing. Therefore, differences in prior exposure and practice are also unlikely to be the cause of the observed inter-subject variability in the outcomes.

The duration of the test was set based on clinical guidelines for the SOT. Although we did not investigate possible adaptation effects in this study, the time-course and magnitude of adaptation for touch elicited via electrical stimulation of the nerve through cuff electrodes are equivalent to natural, mechanically-induced sensations^81^. This suggests that the underlying neural mechanisms for adaptation are similar between mechanically- and electrically-induced sensations. Furthermore, we implemented sensory stimulation that was proportional to pressure underneath the prosthetic foot (i.e., the applied electrical stimulation was modulated based on changes in plantar pressure). Therefore, participants did not receive a constant stimulus, which reduces the likelihood of adaptation due to the dynamic nature of the inputs to the nervous system^82^.

Although participants in this study were transtibial amputees, other populations such as transfemoral amputees and elderly people exhibit comparable sensorimotor characteristics, which predisposes them to an increased risk of fall^2^. It has been reported that when any two sensory inputs are simultaneously compromised in elderly people, a significant increase in sway occurs^52,83^. As such, providing neural sensory stimulation to those who have compromised sensory perception in their lower limbs could be an effective way to improve standing stability in multiple user populations.

## Conclusions

The functional benefits of a sensory neuroprosthesis for improving standing balance were documented by computerized dynamic posturography in two individuals with transtibial limb loss. Appropriately localized and modulated sensations of plantar pressures under the prosthetic foot were elicited by delivering stimulating currents directly to the nerves in the residuum via multi-contact non-penetrating cuff electrodes. We demonstrated these elicited sensations were integrated into the intact neuromuscular control system to reduce sway and increase stability in terms of variations in the Center of Pressure and Equilibrium Scores during perturbed standing. Symmetry of loads applied to the intact and prosthetic legs was also significantly improved with the information provided by the sensory neuroprosthesis. The sensory neuroprosthesis had the strongest impact on maintaining balance when other resources, such as vision, vestibular, or somatosensory inputs from the intact leg, were compromised. These findings indicate that the information provided by a closed-loop sensory neuroprosthesis employing implanted neural stimulation technology was processed by the central and peripheral nervous systems as if they arose from the missing limb to positively impact standing balance. The generalizability of these results on a larger sample of LLAs, and their implications on daily function in uncontrolled home and community environments, their impact on the incidence and risk of falls and losses of balance, and the potential benefits of integrating sensory stimulation with active or semi-active microprocessor controlled prosthetic ankle or knee joints remain to be determined.

## Data Availability

The data that support the findings of this study are available upon request from the corresponding author, H.C.
